# Evaluating the Effectiveness of Tranexamic Acid vs. Placebo in Cardiac Surgery: A Systematic Review and Meta-Analysis

**DOI:** 10.7759/cureus.63089

**Published:** 2024-06-25

**Authors:** Jonathan A Casares, Arturo P Jaramillo, Sajidha Nizamudeen, Angy Valenzuela, Sanod Khan Abdul Samad, Ariana S Rincon Gomez

**Affiliations:** 1 Faculty of Medicine, Pontificia Universidad Católica del Ecuador, Quito, ECU; 2 General Practice, Universidad Estatal de Guayaquil, Machala, ECU; 3 Emergency Department, Travancore Medical College, Kollam, IND; 4 Internal Medicine, Universidad Industrial de Santander, Bucaramanga, COL; 5 General Medicine, Bicol Christian College of Medicine, Legazpi, PHL; 6 Surgery, Universidad Industrial de Santander, Bucaramanga, COL

**Keywords:** cardiac pharmacology, treanaximic acid (txa), antifibrinolytic agent, cardiac surgery, tranexamic acid uses

## Abstract

Tranexamic acid (TXA), a potent antifibrinolytic agent, is widely used in cardiac surgical procedures worldwide to minimize surgical bleeding and reduce the need for perioperative blood transfusions. However, the use of TXA may increase the risk of coronary artery graft thrombosis, potentially leading to a higher occurrence of late thrombotic events. Some studies have suggested that drugs like TXA, aimed at decreasing bleeding during cardiac surgeries, may be associated with elevated risks of thrombotic complications or mortality. Conversely, the reduced need for blood transfusions could contribute to improved long-term outcomes. Thus, a systematic review and meta-analysis were undertaken to assess the efficacy of TXA in cardiac surgery patients. Searches were conducted in databases including PubMed and PubMed Central. Data were extracted, and their quality was assessed using the Cochrane risk of bias tool for randomized clinical trials (RCTs). A random effects model was used to compute the pooled prevalence and investigate heterogeneity using the I2 statistic. Subgroup analyses differentiated between experimental and placebo groups. Additionally, sensitivity analyses were performed to assess the robustness of the findings, and publication bias was examined. An overall sample size of 12,869 patients was included in the meta-analysis, derived from seven of the 10 selected articles. This pooled sample was used to conduct an analysis of TXA's efficacy in cardiac surgery patients. Subgroup analysis revealed a 95% heterogeneity and indicated a p-value of less than 0.05, favoring TXA over placebo in terms of better outcomes. Our research indicates a statistically significant relationship between the efficacy of TXA and the number of patients undergoing heart surgery. According to our findings, there is a pressing need to enhance this evidence base and conduct larger RCTs to better understand the benefits of using TXA, aiming to maintain a low risk of bleeding after both major and minor heart surgeries.

## Introduction and background

Patients undergoing cardiac surgery requiring cardiopulmonary bypass (CPB) often experience significant bleeding and frequently need blood transfusions. Additionally, some may require reoperation due to life-threatening hemorrhage [1]. The use of small blood vessels in adult cardiac surgery increases the risk of renal failure and mortality and also raises the likelihood of bleeding complications in pediatric patients, whose vessels are inherently smaller [2]. Intravenous administration of tranexamic acid (TXA) during cardiac surgery has been shown to significantly reduce postoperative blood loss, thereby decreasing the necessity for blood transfusions [3].

Ethamsylate, a synthetic hemostatic agent, is widely used in various surgeries, including obstetric, orthopedic, and urologic procedures, to minimize blood loss. However, its use in open-heart surgery is uncommon [4-6]. Ethamsylate reduces capillary bleeding by enhancing capillary endothelial resistance and promoting platelet adhesiveness, thereby aiding the primary phase of hemostasis [7]. Both TXA and ethamsylate are readily accessible and cost-effective medications. We hypothesized that the combination of TXA and ethamsylate might be more effective than TXA alone in reducing intra- and postoperative blood loss in pediatric cardiac surgery with CPB [8].

The primary outcome measure was the total blood loss within the first 24 hours post-operation. Secondary outcomes included time to sternal closure, the volume of blood and blood component replacement therapy required, the incidence of postoperative surgical re-exploration, time to extubation, and length of stay in the intensive care unit [9]. Since its introduction in 1962 and particularly after the withdrawal of aprotinin in 2007, TXA has been a primary antifibrinolytic agent used in cardiac surgery [10]. However, several clinical trials have associated high-dose TXA with thrombotic complications and seizures [11].

A randomized clinical trial (RCT) involving 4,631 patients who underwent coronary artery bypass graft surgery demonstrated that TXA reduced bleeding and the need for transfusions without increasing the risk of thrombotic events or death up to one year post-surgery. However, single doses of 100 mg/kg and 50 mg/kg were associated with postoperative seizures, which could potentially lead to stroke and death [12]. Previous research has suggested that a single dose of TXA might be insufficient for patients undergoing prolonged heart surgery [10]. In contrast, continuous infusion of TXA has been shown to maintain a more stable antifibrinolytic plasma concentration with lower peak levels compared with single doses, potentially indicating improved efficacy and reduced side effects [10].

Despite the establishment of TXA delivery methods for high- and low-dose regimens in the 1990s and 2000s through several pharmacokinetic studies, the optimal TXA infusion dose remains controversial due to the limited number of RCTs examining effects on transfusion rates and volumes, postoperative blood loss, and the risks of thrombotic events and seizures [10]. The effectiveness and safety of high-dose TXA infusions were not adequately assessed in these trials due to insufficient participant numbers. Consequently, the Outcome Impact of Different TXA Regimens in Cardiac Surgery with Cardiopulmonary Bypass trial was designed to compare high and low doses of TXA for continuous infusion in patients undergoing cardiac surgery [10]. This multicenter RCT included a one-year follow-up period.

One of the primary objectives of this systematic review and meta-analysis is to thoroughly evaluate the most recent studies on the effectiveness of TXA compared to placebo and other medications used during and after cardiac procedures.

## Review

Methods

We organized our findings into two main sections: the first highlights studies documenting significant reductions in blood loss through the use of TXA in cardiac and other medical procedures; the second deals with studies showing non-significant results.

Record and Search for Studies

This systematic review adhered to the guidelines of the Preferred Reporting Items for Systematic Reviews and Meta-Analyses (PRISMA) [13]. The article selection process involved independent researchers conducting comprehensive searches in PubMed, PubMed Central, and other databases such as Cochrane, Scopus, and Medline. Details of the search methodology employed are found in Table 1.

**Table 1:** Search strategy for databases.

Search Strategy	Databases Used	Number of Papers Identified
Antifibrinolytic agents AND Tranexamic Acid AND Cardiac Surgical Procedures	Pubmed	20
( "Tranexamic Acid/administration and dosage"[Majr:NoExp] OR "Tranexamic Acid/adverse effects"[Majr:NoExp] OR "Tranexamic Acid/analysis"[Majr:NoExp] OR "Tranexamic Acid/pharmacology"[Majr:NoExp] OR "Tranexamic Acid/therapeutic use"[Majr:NoExp] ); AND ( "Cardiac Surgical Procedures/rehabilitation"[Mesh] OR "Cardiac Surgical Procedures/standards"[Mesh] ); OR ( "Antifibrinolytic Agents/administration and dosage"[Majr:NoExp] OR "Antifibrinolytic Agents/pharmacology"[Majr:NoExp]	Pubmed Central	298
"Antifibrinolytic agents [tw]" AND "Tranexamic Acid [tiab]" AND "Cardiac Surgical Procedures [all]"	Others	53

Inclusion and Exclusion Criteria

Two independent authors used the Covidence software to screen the search results from two databases following pre-established inclusion and exclusion criteria, as shown in Table 2.

**Table 2:** Inclusion and exclusion criteria.

Inclusion	Exclusion
Free, full text about probiotic supplementation	Articles that includes pregnant woman
Articles from the past 10 years	Articles from 2013 and below
English-language articles	Non-english studies
Prospective or retrospective studies.	Case reports
Human trials	Animal trials

Data Extraction

During our comprehensive analysis of the research, we observed several significant findings. These include the design of each trial, the number of individuals administered TXA, the characteristics of the placebo group, and the outcomes observed in both the experimental and placebo cohorts.

Risk of Bias Assessment

To determine potential biases in the selected studies, we used the Cochrane risk of bias tool, developed specifically for RCTs. This instrument has gained widespread recognition for its efficiency in assessing the quality of case-series research. The potential for bias in each study was evaluated by reviewers impartially, and any discrepancies in their assessments were resolved through in-depth discussions [14].

Statistical analysis

For all statistical analyses, RevMan version 5.4 (2020; The Cochrane Collaboration, The Nordic Cochrane Centre, Copenhagen, Denmark) was employed. The results of the trials were expressed using the mean difference along with 95% confidence intervals, and an odds ratio effects model was applied for pooling the data. To calculate the standard deviations or standard errors not reported in the trials, we followed the methodology proposed by Mantel-Haenszel et al. Due to the potential high variance arising from diverse study designs and populations, a fixed-effect model was chosen over a random-effect model.

Forest plots were used to visually assess the pooled results. The chi-square test was utilized to identify any discrepancies between the subgroups. Study heterogeneity was quantified using Higgins I2. Publication bias was evaluated through visual examination of the funnel plot, with a significance threshold set at p<0.05.

Results

**Figure 1:**
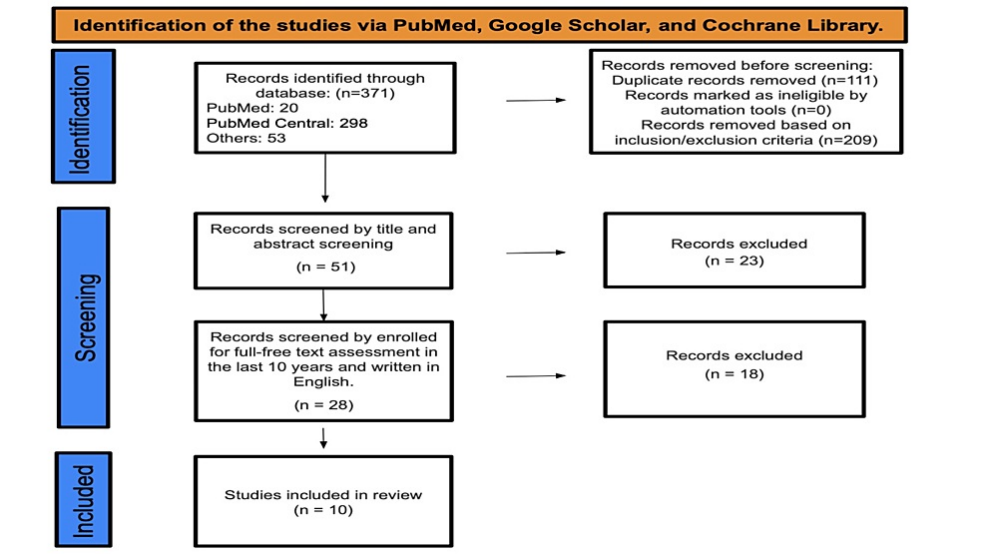
PRISMA diagram. Identification of studies via databases and registers. PRISMA: Preferred Reporting Items for Systematic Reviews and Meta-Analyses.

After searches in PubMed, PubMed Central, and other databases, a total of 371 studies were found. Of these, 209 were deemed ineligible based on inclusion and exclusion criteria, and 111 duplicate studies were removed. Fifty-one studies underwent title and abstract screening, with 23 papers being discarded as they were not related to the purpose of our study. The remaining 28 papers were selected based on their English content and availability for full-text evaluation over the past ten years, leading to the elimination of 18 studies. Subsequently, 8 more studies were discarded due to insufficient numerical data for our RevMan5.4 meta-analysis software. Ultimately, only 10 studies were included in the final data collection (Figure 1).

Table 3 presents an in-depth description of the articles we decided to use. For an evaluation of the risk of bias in randomized controlled trials, see Table 4, which utilizes the Cochrane risk of bias tool.

**Table 3:** Data extraction. RCT: Randomized Clinical Trial; PAI-1: Plasminogen Activator Inhibitor-1; TAFI: Thrombin Activatable Fibrinolysis Inhibitor; PAP: Plasmin AntiPlasmin Complex, also Plasmin-Antiplasmin Complex; tPA: Tissue Plasminogen Activator; TM: Thrombomodulin; TXA: Tranexamic Acid; EACA: Epsilon-Amino-Caproic Acid; AAA: Aortic Abdominal Aneurysm; ICU: Intensive Care Unit; CABG: Coronary Artery Bypass Graft.

Author	Year of publication	Study design	Primary research	Outcome evaluation
El Baser II et al. [9]	2021	RCT	The study examined three groups of 126 children undergoing heart surgery. There were three groups: control (n = 42), TXA (n = 42): got just TXA, and combined ethamsylate (n = 42): received both TXA and ethamsylate.	In juvenile heart surgery, ethamsylate combined with TXA reduced postoperative blood loss and whole blood transfusions more than TXA alone.
Shi J et al. [10]	2022	RCT	Between December 26, 2018, and April 21, 2021, 4 Chinese hospitals recruited 3079 participants for the trial, which ended on May 21, 2021.	The percentage of patients who underwent allogeneic red blood cell transfusion was somewhat reduced by a high-dose TXA infusion.
Zhou ZF et al. [15]	2021	RCT	We randomly assigned thirty heart valve surgery patients to the placebo, low-dose, and high-dose groups 1: 1: 1.	When patients arrived at the ICU and on the first postoperative morning, the low-dose and high-dose groups had lower D-dimers than the control group. Concentrations of PAI-1, TAFI, and TM differed significantly across groups throughout time, but not PAP or tPA (P < 0.05).
Verma S et al. [16]	2020	RCT	Compared to EACA, TXA significantly decreased postoperative bleeding in off-pump CABG at 24 hours.	80 patients with off-pump CABG were randomly assigned to receive TXA, or EACA. We examined the patients for main and secondary outcomes after surgery.
Monaco F et al. [17]	2020	RCT	Randomizing 50 patients per group. TXA, or placebo, was randomly assigned to 100 elective open AAA repair patients.	Open AAA repair with TXA did not decrease intraoperative blood loss or blood transfusions, but it may minimize postoperative blood loss without increasing adverse effects.
Myles PS et al. [18]	2019	RCT	Patients with coronary artery surgery receive aspirin or placebo, as well as TXA or placebo.	The TXA group had 3.8% death or disability at 1 year, whereas the placebo group had 4.4%.
Leff J et al. [19]	2019	RCT	The standard dosage of intraoperative EACA or TXA was compared in 114 cardiopulmonary bypass patients.	Although EACA and TXA had comparable effects on chest tube drainage, EACA is associated with fewer transfusions in CABG procedures.
Zhang Y et al. [20]	2018	RCT	We randomly administered TXA or saline to 210 patients receiving primary and isolated on-pump CABG.	Compared to placebo, TXA reduced allogeneic RBC requirements in terms of volume transfused, ratio exposed, and blood loss volume.
Myles PS et al. [21]	2017	RCT	4662 patients consented, 4631 had surgery and had outcome data, 2311 were allocated to the TXA group, and 2320 to the placebo group.	Within 30 days after coronary-artery surgery, TXA reduced bleeding risk without increasing mortality or thrombotic complications.
Muthialu N et al. [22]	2015	RCT	After heart surgery, 50 children were prospectively randomized to receive aprotinin, or TXA, from September 2009 to February 2010.	Anti-fibrinolytics have a crucial role in open-heart surgery for high-risk children. Without increasing morbidity or death, TXA is as effective as aprotinin.

**Table 4:** Cochrane risk of bias tool.

Studies	Random sequence generation (selection bias)	Allocation concealment (selection bias)	Blinding of participants	Blinding of personnel/care providers (performance bias)	Blinding of outcome assessor (detection bias)	Incomplete outcome data (attrition bias)	Selective reporting (reporting bias)	Other biases	Overall
El Baser II et al. [9]	+	+	+	+	+	+	+	-	7/8
Shi J et al. [10]	+	+	+	+	?	+	+	-	6/8
Zhou ZF et al. [15]	+	+	+	+	?	+	+	-	6/8
Verma S et al. [16]	+	+	+	+	+	+	+	-	7/8
Monaco F et al. [17]	+	+	+	+	?	+	+	-	6/8
Myles PS et al. [18]	+	+	+	+	-	+	+	-	7/8
Leff J et al. [19]	+	+	+	+	?	+	+	-	6/8
Zhang Y et al. [20]	+	+	+	+	+	+	+	-	7/8
Myles PS et al. [21]	+	+	+	+	+	+	+	-	7/8
Muthialu N et al. [22]	+	+	+	+	+	+	+	-	7/8

Meta-analysis of outcomes

**Figure 2:**
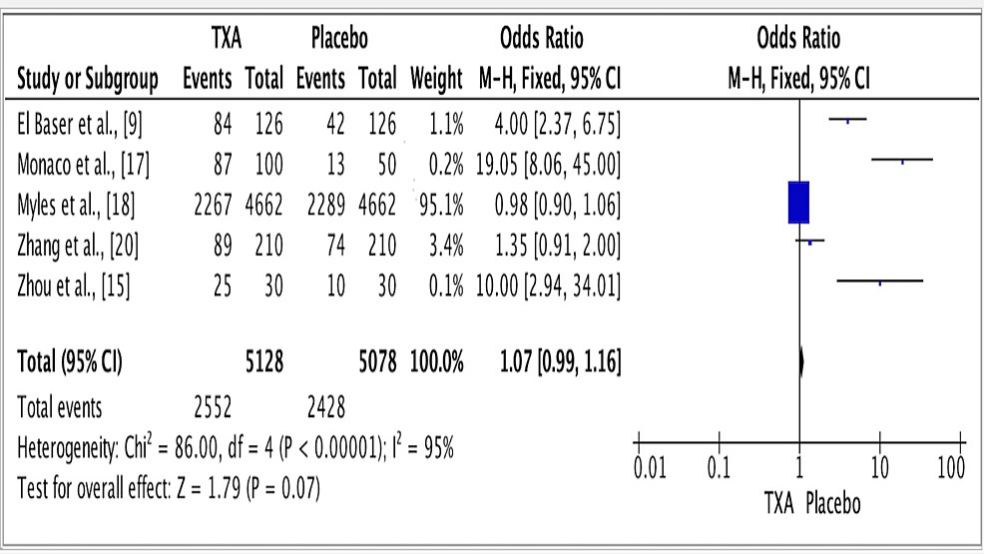
Forest plot for studies about the efficacy of TXA vs. placebo groups. Source: [9,15,17,18,20] TXA: Tranexamic acid.

The results of five studies showed an odds ratio of 1.07 for the efficacy of TXA vs. placebo groups. The odds ratio was 1.07 (fixed effect, 95% CI: 0.99-1.16), the p-value was <0.001, and the heterogeneity (I^2^) was 95% (Figure 2).

**Figure 3:**
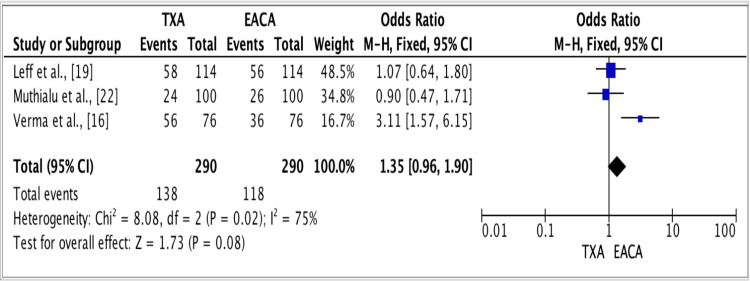
Forest plot for studies about the efficacy of TXA vs. EACA groups. Source: [19,22,16] TXA: Tranexamic acid; EACA: Epsilon-amino-caproic acid.

The results of three studies showed an odds ratio of 1.35 for the efficacy of TXA vs. epsilon-amino-caproic acid (EACA) groups. The odds ratio was 1.35 (fixed effect, 95% CI: 0.96-1.90), the p-value was 0.02, and the heterogeneity (I^2^) was 75% (Figure 3).

**Figure 4:**
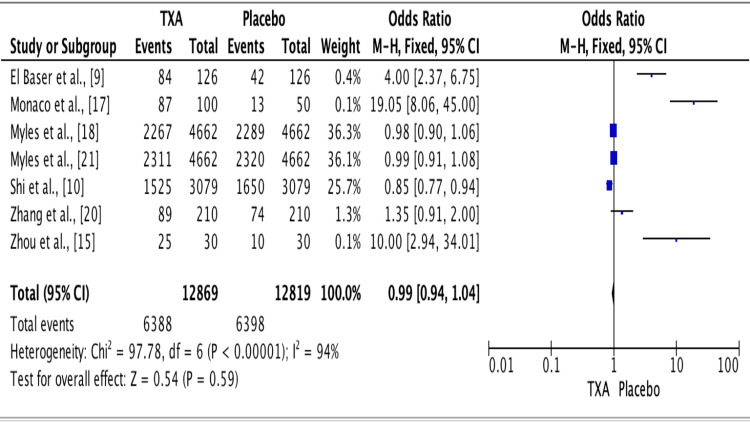
Forest plot for studies about the overall efficacy of TXA vs. placebo groups. Source: [9,10,15,17,18,20,21] TXA: Tranexamic acid.

The results of ten studies showed an odds ratio of 0.99 for the efficacy of TXA vs. placebo groups. The odds ratio was 0.99 (fixed effect, 95% CI: 0.94-1.04), the p-value was <0.001, and the heterogeneity (I^2^) was 94% (Figure 4).

**Figure 5:**
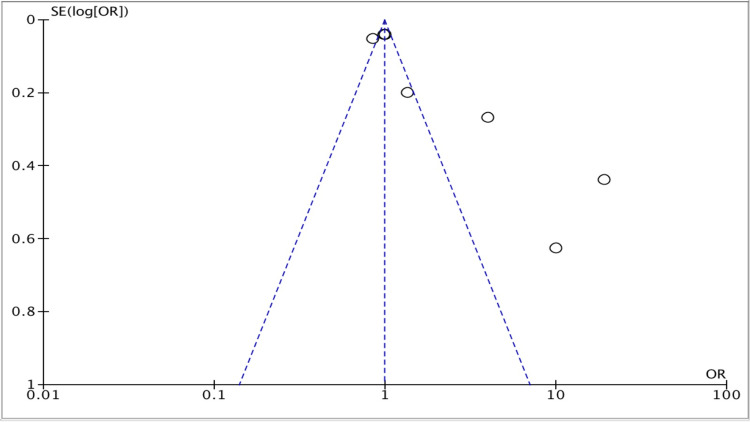
Funnel plot for all included studies about the efficacy of TXA vs. placebo groups. Source: [9,10,15,17,18,20,21] TXA: Tranexamic acid.

Publication bias was observed in three of the studies (Figure 5).

Discussion

TXA is heralded for its remarkable ability to minimize blood loss across a variety of surgical interventions, significantly improving survival rates among patients in obstetric and trauma settings who face severe bleeding episodes [10]. Primarily functioning as a fibrinolysis inhibitor, TXA also offers anti-inflammatory benefits, potentially reducing the systemic inflammatory responses observed in certain cardiac surgery patients. However, balancing its benefits with potential risks, such as seizures and other adverse effects which could increase healthcare costs, necessitates a standardized approach to TXA administration.

The drug is generally well-received, with most adverse reactions being mild to moderate in nature; severe side effects are rare, corroborated by clinical trials and literature confirming its safety across numerous surgical scenarios. Our detailed review integrates findings from diverse studies, presenting a comprehensive analysis of current research.

In a pivotal study by Shi J et al., researchers investigated the impact of low-dose versus high-dose TXA on fibrinolysis in adults undergoing low-risk valvular cardiac surgery via cardiopulmonary bypass [10]. Their results supported earlier findings that low-dose TXA is as effective as high-dose regimens, although concerns about the safety of higher doses were raised due to increased thrombomodulin production [10]. A significant reduction in D-dimer levels was noted among patients treated with either dose upon their arrival in the ICU and on the following morning, compared to those in the placebo group [10]. In a study by El Baser II et al., the combination of ethamsylate and TXA in pediatric cardiac surgeries proved more effective in reducing sternotomy closure times, postoperative blood loss, and overall transfusion requirements than TXA alone [9], though it did not significantly alter the need for intra- and postoperative blood products [9].

Verma S et al. assessed the effectiveness of TXA and EACA in reducing postoperative blood loss in off-pump coronary artery bypass grafting [16]. Their findings indicated no substantial differences in bleeding at four hours post-operation, but after 24 hours, the TXA group showed significantly less bleeding compared to the EACA group [16]. Similarly, Karski JM et al. observed that TXA was far more potent than aminocaproic acid in reducing blood loss [23]. Conversely, Monaco F et al. reported no significant reduction in intraoperative blood loss during open abdominal aortic aneurysm repairs, although they noted a reduction in postoperative bleeding [17], suggesting TXA's underrecognized potential [17].

Over a longer term, Myles PS et al. explored the outcomes of TXA in coronary artery surgeries, noting no reduction in death or disability rates at the one-year mark [18]. Although no significant interaction was found between patients who had taken aspirin on the day of surgery and those who hadn’t, subtle indications of TXA's potential beneficial effects were noted [18]. Leff J et al.'s trial found EACA to significantly lower transfusion rates compared to TXA within the first 24 hours after surgery, though no significant differences were observed in other outcomes [19].

Zhang Y et al. demonstrated that TXA significantly reduced postoperative bleeding and the necessity for allogeneic transfusions in primary and isolated CABG surgeries without negatively impacting morbidity or mortality [20]. This study also highlighted the long-term benefits of TXA, showing a decreased incidence of myocardial infarction over a seven-year follow-up [20]. Similarly, evidence from Myles PS et al. suggested that TXA does not increase the risk of death or thrombotic complications after coronary-artery surgery [21]. Additionally, Muthialu N et al. linked TXA to a reduced risk of bleeding complications relative to placebo, despite an increased risk of postoperative seizures [22], affirming TXA’s efficacy and safety in high-risk pediatric cardiac procedures [22].

Limitations

While subgroup analyses can provide insights into the effects of TXA in different segments of the population or under various conditions, they are often limited by the power and data availability, and the results can sometimes be speculative rather than definitive. Other limitations include that the review may not address whether the effects of TXA vary according to different dosages, as studies included in the analysis might use different dosing regimens.

## Conclusions

Our comprehensive analysis has shown that both in pediatric and adult cardiac surgeries, such as abdominal aortic aneurysm repairs, cardiac valve replacements, and coronary artery bypass grafting (CABG), trials comparing TXA and EACA to placebo have yielded positive outcomes. Similar to other studies, the difference between TXA and EACA when compared to placebo is not statistically significant. However, TXA demonstrates slightly better outcomes than EACA. This is why we advocate for more RCTs to clarify the dosage per kilogram more straightforwardly and to establish criteria or guidelines for when TXA is useful and when it is not. While these results are promising, it is critical to maintain a balanced perspective. Because antifibrinolytics, dosages, and study designs vary, further research is needed to definitively prove that TXA is safe, effective, and offers long-term benefits during cardiac procedures. Therefore, we support more thorough, large-scale RCTs to confirm these preliminary findings and to determine the true potential of TXA as a treatment in both major and minor heart surgeries.
